# Computational Study of Synthetic Agonist Ligands of Ionotropic Glutamate Receptors

**DOI:** 10.1371/journal.pone.0058774

**Published:** 2013-03-25

**Authors:** Tino Wolter, Thomas Steinbrecher, Marcus Elstner

**Affiliations:** Department of Theoretical Chemical Biology, Institute for Physical Chemistry, Karlsruhe Institute of Technology, Karlsruhe, Germany; Medical School of Hannover, United States of America

## Abstract

Neurological glutamate receptors are among the most important and intensely studied protein ligand binding systems in humans. They are crucial for the functioning of the central nervous system and involved in a variety of pathologies. Apart from the neurotransmitter glutamate, several artificial, agonistic and antagonistic ligands are known. Of particular interest here are novel photoswitchable agonists that would open the field of optogenetics to glutamate receptors. The receptor proteins are complex, membrane-bound multidomain oligomers that undergo large scale functional conformational changes, making detailed studies of their atomic structure challenging. Therefore, a thorough understanding of the microscopic details of ligand binding and receptor activation remains elusive in many cases. This topic has been successfully addressed by theoretical studies in the past and in this paper, we present extensive molecular dynamics simulation and free energy calculation results on the binding of AMPA and an AMPA derivative, which is the basis for designing light-sensitive ligands. We provide a two-step model for ligand binding domain activation and predict binding free energies for novel compounds in good agreement to experimental observations.

## Introduction

Glutamate is the most important excitatory neurotransmitter of the mammalian central nervous system. Its interactions with cellular receptor proteins are therefore of highest interest for our understanding of neurological function and pathology on the molecular level. The responding receptors are divided into two classes, the metabotropic and ionotropic glutamate receptors, or mGluR and iGluR. The former are a type of G-protein coupled receptor, the latter are ligand gated cation channels which are essential for the fast synaptic transmissions between nerve cells.

iGluRs are further subdivided into three families depending on their sensitivity to different agonists, which are α-amino-3-hydroxy-5-methyl-4-isoxazole-propionic acid (AMPA), (2S-3S,4S)-3-(carboxymethyl)-4-prop-1-en-2-ylpyrrolidine-2-carboxylic acid (kainate) and N-methyl-D-aspartate (NMDA) [1], [2].

The iGlu receptors play a fundamental role in neuronal function and development, e.g. learning and memory [3], [4]. Furthermore, iGluRs are associated with several neurological disorders, like epilepsy, schizophrenia, Alzheimer’s disease and Parkinson’s disease [5], [6]. These roles make them interesting not only for fundamental biochemistry but a better understanding of their structure and function would be of great pharmaceutical significance as well.

Structurally, ionotropic glutamate receptors are typically tetra or –pentamers. Each subunit is composed of an extracellular N-terminal domain (ATD), at least one extracellular ligand binding domain (LBD), a channel-forming transmembrane domain (TMD) and a cytoplasmic C-terminal domain involved in signaling. The binding of glutamate or synthetic agonists to the LBD induces the opening of the channel. Partial agonists that result in very low channel activity are also known. In the following, we will focus on the AMPA sensitive iGluR2 and specifically the process of agonist binding to its LBD.

The iGluR LDB is composed of two fairly rigid subdomains structured like two halves of a clamshell. They are known to undergo a transition between an open and a closed state while binding a ligand [7]. A closing of the LBD then causes an opening of the cation channel. To investigate the mechanism of the glutamate receptors a lot of work has focused on the isolated, soluble LBD (S1S2) which can be over-expressed in bacteria.

A wide range of LBD X-ray crystal structures are available, including ones that are co-crystalized with agonists, partial agonists and antagonists [8]–[12]. The LBD dynamics have been analyzed by NMR [13]–[17], fluorescence [18], [19] and infrared spectroscopy [20]–[22]. Nevertheless, many questions about the details of ligand binding and channel activation remain. A correlation of degree of domain closure and activity was established from X-ray structure analysis. The proposed activation mechanism suggests that a full agonist closes the clamshell completely, while a partial agonist would result in incomplete closure [8]. See Figure 8 in Ref. [8] for a schematic representation of the receptor organisation and structural changes in the different activation states.

However, not all observations fit into this model, e.g. the ligand AMPA shows only partial agonistic behavior when acting on the L650T mutant of the iGluR2 domain while still fully closing the receptor. For this ligand, X-ray crystal structures showing evidence both for a closed and a partially closed state have been reported. The latter was also suggested to represent the inactive state of the receptor [23]. There are several more cases in which a full receptor closing is accompanied by partial agonistic behavior [24]–[26]. A structural analysis of two partial agonists showed identical domain closure for both ligands while the corresponding electrophysiological measurements gave considerably different activation potentials, which was explained by a twist of domain 2 [27].

These open questions regarding the molecular details of the iGluR LBD function is particularly well suited to be addressed by molecular simulation methods. However, due to the large system size and macromolecular conformational changes involved, this problem has only recently been addressed by theoreticians.

One theoretical study investigated different crystal structures and described molecular dynamics via principal component analysis. It showed that three important eigenvectors capture the main receptor motion, these were called the bending mode, the twisting mode and the rocking mode [28]. Various molecular dynamics simulation studies have been conducted to investigate the internal dynamics of the LDB to give an inside into the function of the glutamate receptor [29]–[34]. Recently, thermodynamic properties concerning the binding of several ligands have been obtained by extensive free energy calculations [35], [36].

**Figure 1:**
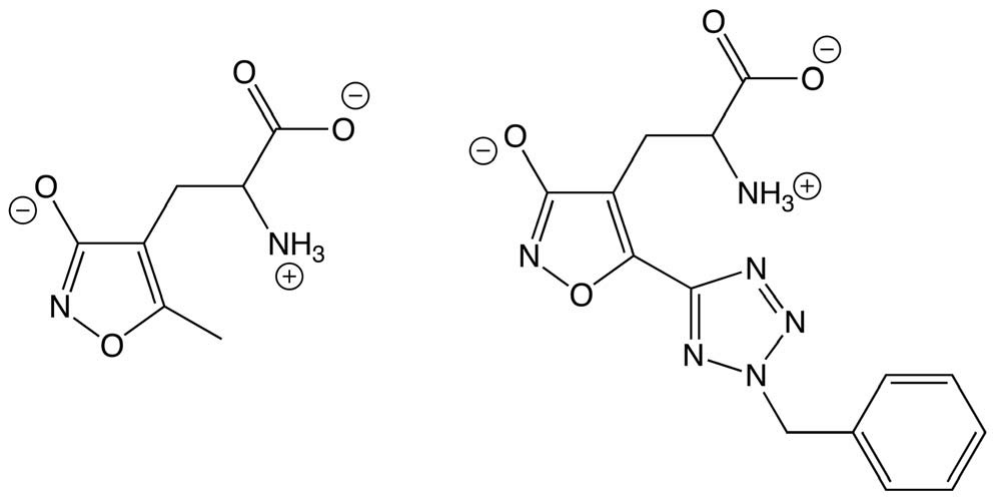
Chemical structures of AMPA (left) and 2-BnTetAMPA (right) shown in their zwitterionic state.

In this paper we will first present a comprehensive view on the binding process of AMPA. Using two different free energy methods we show that ligand binding properties can be calculated from very long umbrella sampling simulations with no further artificial system constraints. In contrast, Metadynamics is a promising novel free energy calculation method that despite minor convergence problems works well for a system of this complexity and size. Our benchmark results for the well-studied AMPA ligand also serve to validate calculations on a novel iGluR ligand, 2-BnTetAMPA (BTA). See Figure 1 for chemical structures of the AMPA and BTA ligands. This compound is an AMPA derivative, that is a key compound in designing photo-switchable ligands i.e. ligands that undergo light-induced conformational change.

BTA-based ligands and their binding to iGluR LBDs are of particular interest since glutamate receptors have become a focus of the emerging field of optogenetics [37]. Optogenetics refers to the manipulation of genomes and proteomes by making them light-sensitive and offers exciting prospects both for basic and applied life science studies. In recent years, particularly the development of photo-switchable ligands for ion channels, e.g. glutamate receptors, has revolutionized the study of such receptors by introducing easily triggered on/off switches [37]–[42]. Since the microscopic connection between molecular geometry changes, binding mode and affinity differences and macromolecular function are still mostly unclear, we aim at providing an atomistic model of this process.

## Methods

All molecular dynamics (MD) simulations used X-ray crystal structures for initial model building. Experimental binding mode information was available for both studied ligands. The AMPA and BTA complex structures (pdb-code 1FTM [8] and 2P2A [9]) were used for the bound states and the apo-protein structure was built from the crystal structure with pdb-code 1FTO [8]. To ensure computational efficiency and equal size for all simulated systems, only a single LBD was selected in each case.

Protein structures were completed by automatic model building tools, embedded in cubic periodic boxes of 9.7 nm side lengths, solvated with ca. 30,000 TIP3P [43] water molecules and neutralized by adding chloride anions. The Amber99SB force field [44] was used to describe the LBD and ligands were parameterized according to the gaff force field [45] using the Antechamber module of Amber Tools version 11.

All systems were subjected to an equilibration procedure consisting of 500 steps of steepest descent minimization, followed by 500 ps of temperature and volume equilibration to 300 K and average system densities of 0.98 g/ml. The Nose-Hoover thermostat [46] and Parrinello-Rahman barostat [47] were used throughout. During equilibration, the protein structure was restrained by harmonic forces of 1000 kJ mol^−1^ nm^−2^. All simulations were performed using the Gromacs simulation package version 4.5.5 [48]. To conduct Metadynamics simulations, the PLUMED-plugin version 1.3 [49] was used.

Free energy calculations were conducted using either umbrella sampling (US) techniques or the Metadynamics approach. US is a well established method to calculate the potential of mean force along a predefined reaction coordinate [50]. The use of harmonic biasing potentials ensures even sampling along the reaction coordinate, even in high-energy barrier regions. US simulations have been used successfully in a variety of molecular simulation studies in the past [51]–[54] and are known to perform best if the defined reaction coordinate is well-suited to the studied process, i.e. is close to the correct minimum free energy pathway. Even a completely nonphysical enforced reaction pathway would give correct information about relative end state free energies while overestimating barriers, but may make it much harder to obtain converged results. Free energy data was analyzed using the Gromacs g_wham tool and custom made analysis programs.

Metadynamics simulations use a similar approach, but does not require a predefined reaction coordinate. Instead, the system phase space trajectory is projected onto a set of collective variables (CV). At given time intervalls, gaussian-shaped biasing potentials are automatically added to the total potential function, depending on the current position of the system in CV space. Over long simulation times, the sum of all biasing potentials should approach the negative potential energy surface (PES) of the system. Metadynamics history-dependent biasing potential approach offers some attractive characteristics over traditional free energy techniques. The system is assumed to automatically explore minimum energy pathways between stable minima and sequential ‘filling’ of minima should prevent the system from entering thermodynamic trap states. Towards the ends of a Metadynamics simulation, the PES including the biasing potentials will be very flat, leading to highly efficient sampling. The approach has been successfully applied to biochemical problems before [55]–[58], but it was found that the efficiency of a Metadynamics simulation will strongly depend on selecting a suitable set of CV and biasing potential parameters. The intricate potential energy landscapes of macromolecular complexes are known to be among the more difficult systems to describe with any enhanced sampling approach.

## Results

### Free MD Simulations

Before performing free energy calculations on the complexes, initial unrestrained molecular dynamics simulations of 200 ns length have been conducted for all three systems under study, the receptor-AMPA complex, the receptor-BTA complex and the apo-protein, to investigate their structural properties and to remove conformational deformations from X-ray crystallization artifacts. As starting structures the preequilibrated systems as described above were used.

**Figure 2:**
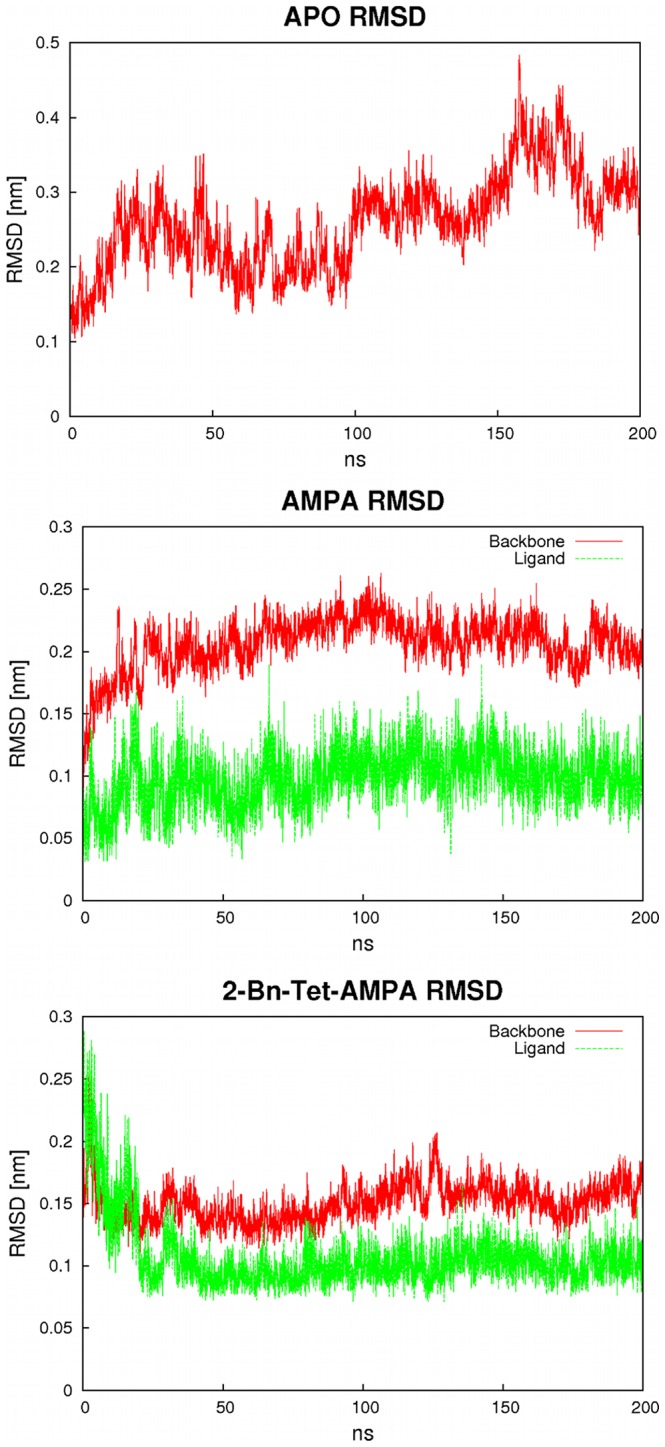
RMSD-values for the protein backbone (red) from 300 ns length MD simulations of the apo-protein (top), AMPA complex (middle) and BTA complex (bottom). For the complexes, the corresponding ligand RMSD-values are depicted as green curves.

In these simulations, we see a high degree of stability for both closed protein ligand complexes, with root mean square deviation (RMSD)-values in the range of 0.2 nm (AMPA) and 0.15 nm (BTA) (see Figure 2). Both complexes exhibit no large structural changes between 20–200 ns, indicating successful equilibration. Likewise, comparable side chain fluctuations are found for both complexes, with low fluctuations of ca. 0.05 nm in domain 1, with higher values for the terminus and flexible loops around residues 21 and 63. For domain 2, slightly higher fluctuations in the 0.1–0.15 nm range are found. The degree of closure of the clamshell domain structure is about 100 degrees for either system, no signs of spontaneous receptor cleft opening were found. Ligand RMSD-values in the range of 0.1 nm indicate that for both AMPA and BTA, the ligand binding mode remains virtually unchanged over the course of the simulation. Overall, the closed receptor with a bound ligand appears to form a stable binding geometry that is not noticeably perturbed by the use of the LBD monomer in our simulations instead of the complete receptor.

**Figure 3:**
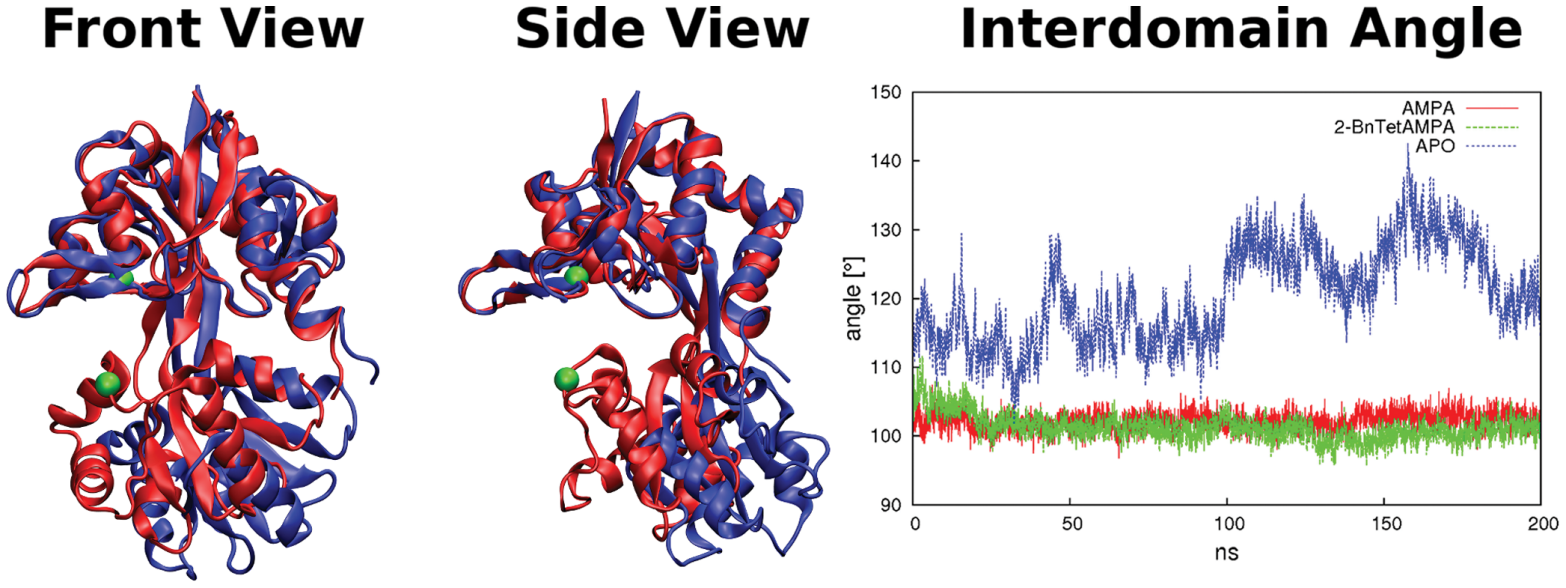
Comparison of apo-protein X-ray crystal structure (red, pdb code 1FTO) and the structure of maximum interdomain angle from the end of a 300 ns MD simulation (blue). Left: view from the front; middle: from the side. A wider opening of the LBD in MD compared to the crystal structure is observed. The two amino acid residues used to defined the opening reaction coordinate are shown in green. Right: Interdomain angle for the three simulations. Complex angles remain at ca 112 degrees compared to 140 degrees for the apo structure. To compute the interdomain angle, the domains have been defined as residues 394–495 and 732–771 for domain 1 and residues 500–728 for domain 2, connected by a flexible hinge comprising residues 496–499 and 729–731.

The simulation of the open apo-protein yields somewhat higher RMSD-values that do not reach a stable value over 200 ns of MD simulation but rise to 0.3 nm and above. Amino acid residue fluctuations are similarly increased to ca. 0.2 nm and no higher stability for domain 1 can be found. The structural change of the apo-protein corresponds to the receptor opening wider than seen in the X-ray structure, to 140 degrees (see Figure 3). The conformational change occurs mostly by movement along the first two eigenvectors (clamshell motion and twisting mode, see below). The apo-protein remains in the open conformation with a wider opening angle possibly caused by the lack of inter-subunit contacts in our monomer simulation.

**Figure 4:**
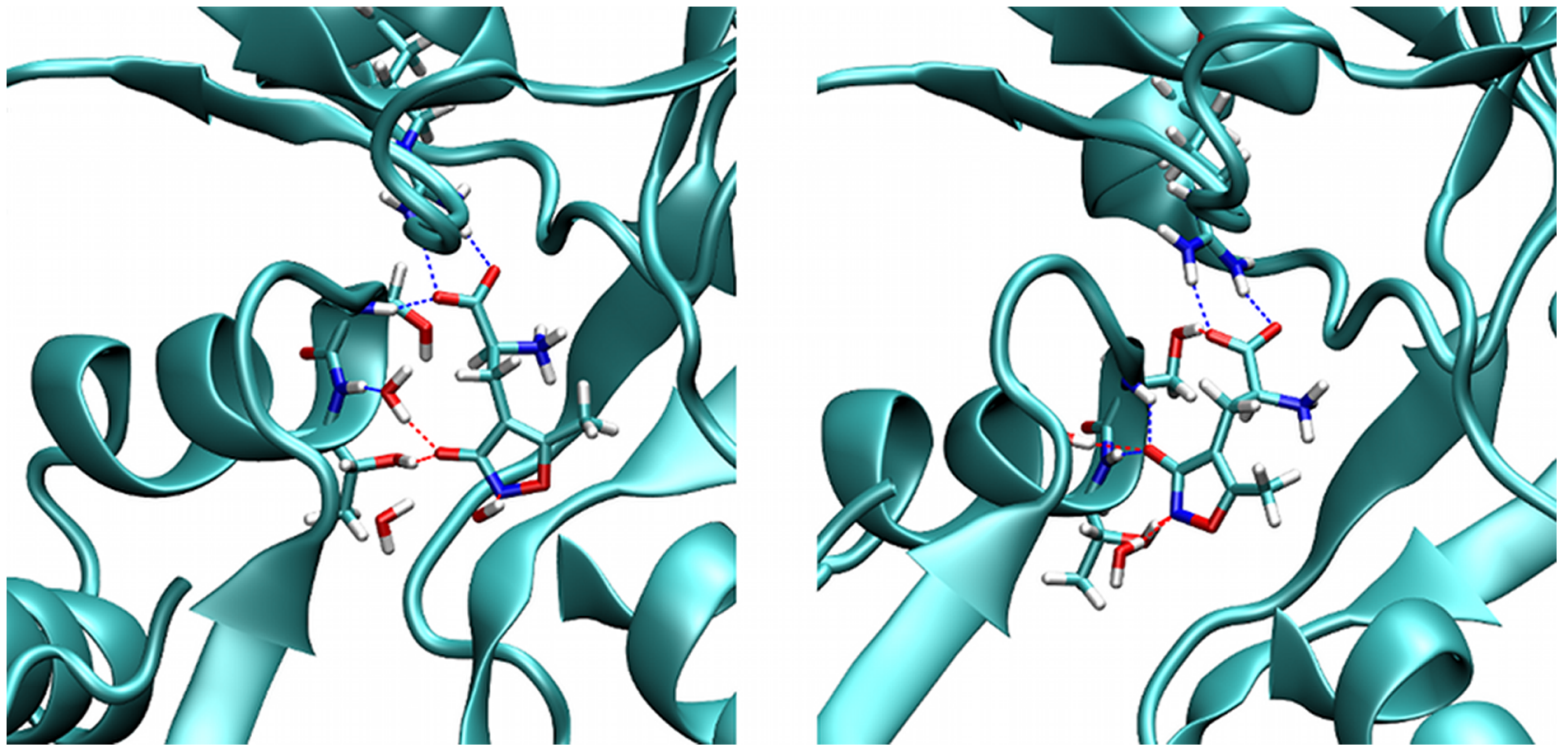
Rearrangement of the AMPA-LBD hydrogen bonding network. At the start of the MD simulations, the AMPA-Thr655 hydrogen bond is water mediated (left), but after water dissociates out of the binding site it is replaced by a direct hydrogen bond (right). This is accompanied by a change of the AMPA-Ser654 backbone hydrogen bond into an AMPA-Ser654 sidechain one. The main ligand-LBD interaction between the AMPA-carboxylate and Arg485 is maintained. Shown is the part of the protein structure comprising the binding site (cyan cartoon representation), the ligand colored by element type in stick representation and crucial receptor amino acid sidechains interacting with the ligand.

Regarding the ligand binding mode, we observe a rearrangement of water molecules in the AMPA-complex. In the X-ray structure, the ligand interacts with the domain 2 residue Thr655 backbone NH-group via a bridging water molecule. This water molecule disengages from the complex during free MD simulations and already after 50 ps of simulation time, the hydrogen bonding network has shifted slightly (see Figure 4). The 3-hydroxy-oxygen of AMPA now directly interacts with both Thr655 and Ser654 backbone functional groups. This shift is accompanied by a slight rearrangement of the AMPA carboxylate group that replaces a hydrogen bond to the backbone of Ser654 with one to the Ser654 side chain hydroxyl group. The AMPA carboxylate group maintains its functionally important salt bridge to Arg485, a main feature of all iGluR2 ligands. The displacement of water mediated hydrogen bonds by direct ones has not been described in previous MD simulation reports of this system. We have repeated the model building and equilibration steps of the complex using the alternative TIP4P and TIP5P water models, and the same change in the hydrogen bonding network occured on similar time scales. This feature of our model will be discussed in more detail below. In general, as evident by the ligand RMSD-value of 0.1 nm, the overall binding mode of AMPA is not significantly affected by this change in binding mode. For the BTA ligand, no comparable rearrangement can be observed, since BTA adopts a glutamate-like binding mode in which the negatively charged oxygen atom interacts directly with Thr655 backbone NH-group.

### Rigid Body PCA

To obtain a simplified picture of the major conformational motions of the simulated systems, we have performed principal component analysis on each of the three 200 ns MD simulation trajectories. To focus specifically on ligand binding/unbinding phenomena, we describe the receptor as composed of two rigid domains (defined as residues 394–495, 732–771 for domain 1 and residues 500–728 for domain 2) connected by a flexible hinge (residues 496–499,729–731), as in previous studies [28], [35], [36]. To remove internal motion of the two rigid domains, their optimized structure was superimposed onto the corresponding residues of the MD trajectory.

**Figure 5:**
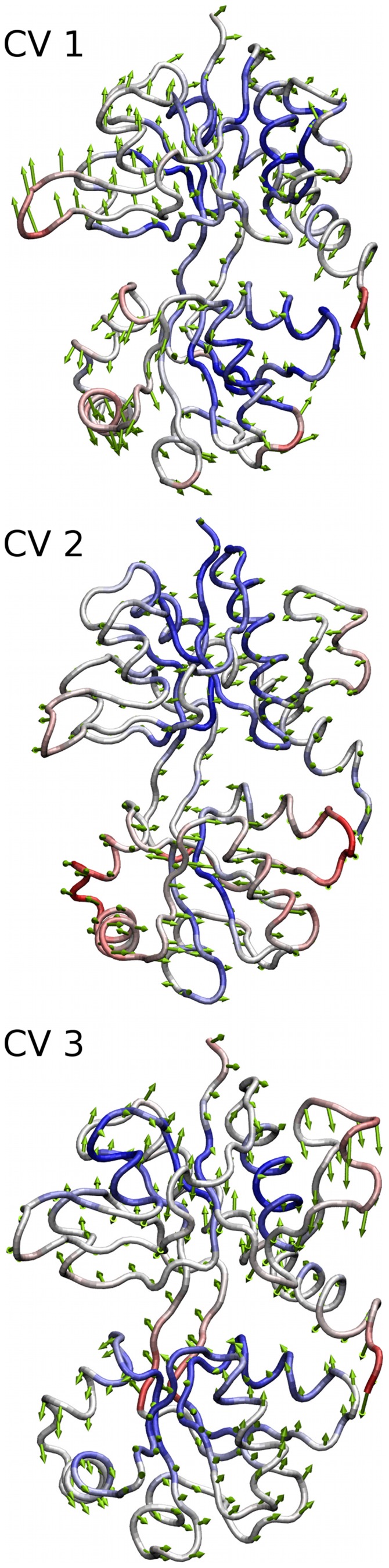
Arrow representation for the three main eigenvectors of iGlu2 receptor conformational motion. The three modes depicted are labeled clamshell (CV1), twisting (CV2) and rocking (CV3) motion, respectively. For a better visualization of the eigenmodes in questions, we have added additional animated movies depicting the conformational changes. See Supplementary Material for Movies S1, S2 and S3.

For both complexes as well as the apo-protein, we find three dominant eigenvectors which we term clamshell, twisting and rocking motion, in accordance with previous work based on X-ray crystal structures [28] (see Figure 5, arrow representation). The similarity of the PCA results for the protein-ligand complexes and the apo-protein show that the fundamental dynamics of the receptor remain unchanged with either ligand bound and are independent of the starting conformation (i.e. open or closed form). Therefore, our equilibrated models of the complexes and the apo-protein serve as good representations of the start and end points of the functional receptor opening/closing conformational change.

The three dominant eigenvectors obtained from the rigid body PCA were then used as the collective variables for metadynamics simulations, as described below.

### Binding Free Energies via Umbrella Sampling

We have determined the binding free energy of AMPA and BTA to the iGlu2 receptor via umbrella sampling simulations. For the case of AMPA, a previous study used free energy calculations based on umbrella sampling biasing in combination with additional orientational restraints [36]. We present here a more straightforward approach, in which the total binding (or unbinding) process is decomposed into only two sequential steps: I) the opening of the initially closed receptor and II) the removal of the ligand from the open receptor binding site. Apart from the umbrella potentials biasing the position along the reaction coordinate, no further restraints were applied in the system, i.e. we aim for a binding/unbinding trajectory that is as close to natural as possible.

#### Opening/Closing transition of the receptor

For the first substep of opening the receptor, the reaction coordinate was defined as the center-of-mass distance between the backbone atoms of Gly451 and Ser652, two amino acid residues located close to each other, opposite of the hinge region at the domain interface. When the open and closed receptor crystal structures are compared, this distance changes between 0.54 and 1.30 nm. corresponding to a change in the interdomain angle from 100° to 120°. For a more detailed analysis of how the open and closed states of the receptor correspond to the interdomain motions identified in PCA calculations above, we have analysed the time series of these parameter for different receptor states. See Supplementary Materials for Figures S1 and S2.

We have sampled this reaction coordinate from 0.5 nm to 1.4 nm in our simulations in order to fully describe the complete conformational change. The local receptor structure around the residues defining the reaction coordinate was monitored throughout the simulation and found to contain no restraint induced deformation. The pulling simulations to generate starting geometries were conducted using a very small pulling speed (10^−5^nm/ps) to avoid unphysical distortions within the protein structure.

**Figure 6:**
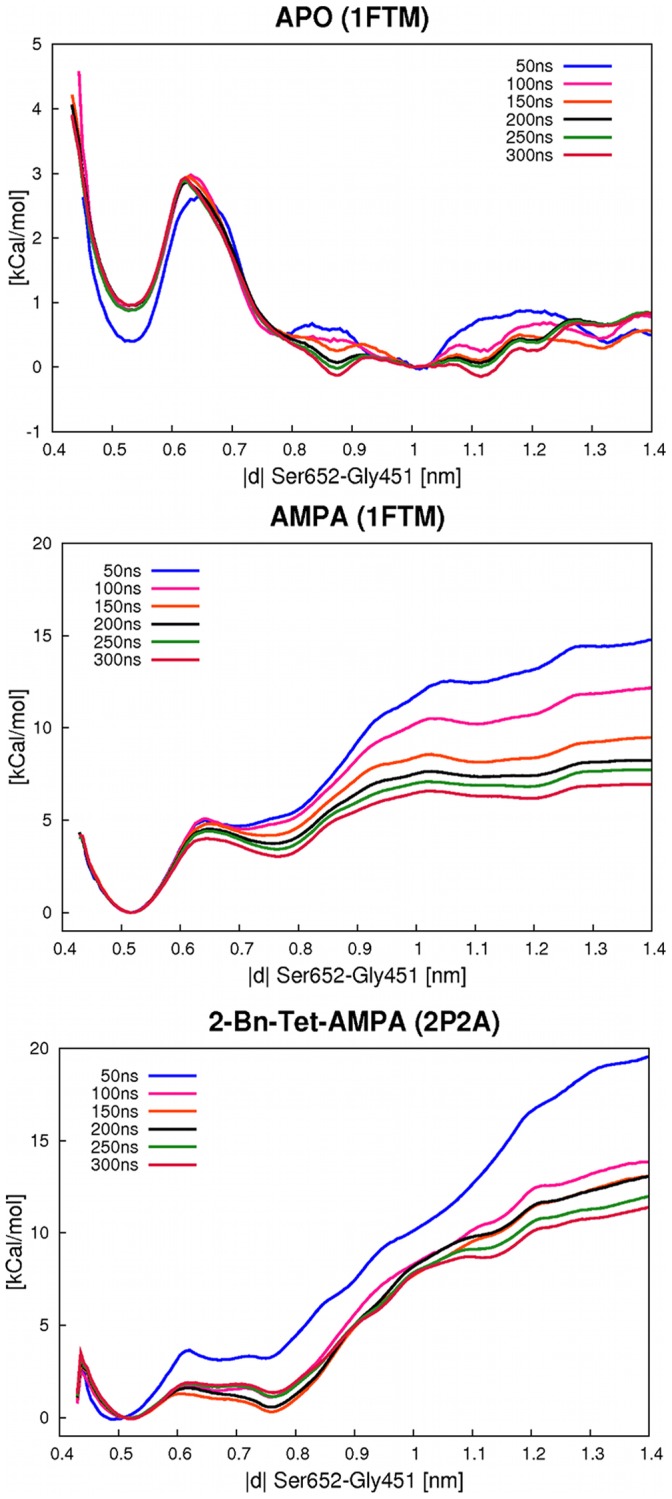
PMF free energy curves for the opening/closing motion of the LBD. The different free energy curves correspond to sequential 50 ns parts of the per-window total 300 ns simulations. Depicted are the curves for the apo-protein (top), AMPA complex (middle) and BTA complex (bottom). The reaction coordinate is defined as the distance between the backbone center-of-mass of Gly451 and Ser652. For all three simulations, a minimum corresponding to the closed state at ca. 0.5 nm is found. For the two complexes, a second minimum at ca. 0.8 nm corresponding to the half-closed receptor state is observed. For the open state, flat free energy plateaus instead of harmonic minima are found, since the LBD is free to open very wide in our monomeric simulations.

A first umbrella sampling simulation was conducted using the ligand free receptor in the open conformation (structure taken from pdb structure 1FTO). We used 20 US windows spaced equidistant at 0.05 nm intervalls, with a 500 kcal mol^−1^ nm^−2^ biasing force constant. For each simulation window an MD simulation of 300 ns length was conducted. Simulation convergence was judged by histogram overlap and by batch averaging over 50 ns simulation intervalls. For the opening process of the ligand free receptor we see a clear local energy minimum at +1.10 kcal/mol and 0.53 nm, corresponding to the closed form of the receptor (see Figure 6). For the open form in the range of 1.0–1.4 nm, a broad free energy plateau instead of a clear minimum is found. Our results show that the open form is more stable by ca. one kcal/mol, indicating that it is the preferred conformation of the free receptor in solution. With respect to convergence with simulation time, we see that the potential of mean force (PMF) shape remains consistent after about 150 ns, but the open form PMF is subject to significant noise and slow to converge. These results indicate that the two step process defined above is a realistic description of ligand binding to iGluR and no additional substep of closing the binding site after removing the ligand is necessary. Our model of the linked equilibrium between receptor opening/closing and ligand binding described above assumes that the open state of the iGluR is the dominant form encountered by solvated ligands. While it can not be ruled out that the dynamic equilibrium of the closed and open apo-receptor does play a role in ligand binding, the fact that we find the open form to be the preferred ligand-free state in solution indicates that our model of ligand binding to the open form, followed by domain closure, is supported by the calculations.

A similar US free energy calculation for opening the ligand binding domain of the AMPA and BTA complexes was conducted with the same setup and reaction coordinate as for the apo-protein above, but with the sampling direction from the closed to the open form of the receptor in this case. Starting structures were based on the pdb X-ray crystal structures 1FTM and 2P2A, respectively. Good overlap of simulation windows was judged by histogram analysis and simulation convergence by 50 ns length batch averages.

For the two ligands, markedly different free energy curves are obtained (see Figure 6). The PMF for opening the AMPA complex shows a broad energy minimum at around 0.51 nm, separated by an energy barrier of ca. 5 kcal/mol from a second, shallower minimum at 0.75 nm. This second minimum, 3.09 kcal/mol higher than the first, then leads to a flattened PMF at distances of ca. 1 nm and a barely discernible, very shallow minimum between 1.1 and 1.2 nm, which we identify as the open state. For this final plateau, extensive sampling is required to obtain its corresponding free energy, as the PMF curves after 50, 100 and 150 ns simulation lengths still show significantly higher energies. After 250 ns of simulation time, the resulting PMF changes only slightly over the last 50 ns and convergence is sufficient to calculate the free energy of opening the AMPA complex as +6.18 kcal/mol.

The three states identified in our simulations, corresponding to the first and second PMF minimum and third PMF minimum/plateau are, respectively, the closed form of the complex, the half-closed form and the open state. This is seen from the RMSD-values in Table 1 in which the observed minima are compared to the various receptor states. For the closed state, the X-ray crystal structure of the AMPA or BTA complex was used, for the half-closed state that can be observed with a bound partial agonist the X-ray crystal structure with pdb code 1MQG was used and for the open state that of X-ray crystal structure 1FTO. Since US simulations of complexes and apo-receptor were initiated from models based on different X-ray crystal structures, there is a possibility of introducing starting structure bias. Table 1 shows this not to be the case, as for any simulation, independent of the starting conformation, the observed minima are closest in structure to the X-ray structure of the corresponding state.

**Table 1:** RMSD-values in nm of receptor conformations and observed PMF minima.

	AMPA			BTA		
Receptor	1st minimum	2nd minimum	3rd minimum	1st minimum	2nd minimum	3rd minimum
closed	0.11	0.14	0.23	0.13	0.15	0.29
half-closed	0.20	0.13	0.20	0.18	0.13	0.26
open	0.31	0.26	0.17	0.29	0.25	0.15

The X-ray crystal structures used for the closed, half-closed and open states had pdb identifiers 1FTM/2P2A, 1MQG and 1FTO, respectively. A clear correspondence of the observed minima and the receptor conformational states is found. Furthermore, the closed and half-closed receptor states appear consistently more similar to each other than either is to the open state, in good agreement to expectations.

The PMF for opening the LBD with a bound BTA ligand (Figure 6) shows a clear minimum corresponding to the closed state at 0.53 nm as well. This is connected by a much lower barrier of ca. 2 kcal/mol height to a second minimum at 0.76 nm. This second minimum is comparably low in energy to the first (+1.35 kcal/mol), unlike that for the AMPA complex. Beyond 0.8 nm, the PMF rises significantly, until a plateau around 1.1 nm and ca. 8 kcal/mol is reached. Again a third, very shallow minimum around 1.12 nm can be postulated, but its exact extent is hard to distinguish, as in the case of the AMPA complex. Overall, a free energy change of +8.69 kcal/mol can be calculated for the opening of the BTA-LBD complex. The three minima correspond well to the expected closed, half-closed and open states of the receptor (see Table 1). Since the open state plateau is not perfectly flat even after 300 ns, there is a somewhat higher inaccuracy in the free energy estimate of opening this complex.

Both in the case of AMPA and BTA, the ligands remain close to their original binding position during the opening process. They remain attached to domain 1 via the Arg485 anchoring group.

Overall, the PMF curves obtained for both complexes agree well with the description of a receptor that has three distinct states. We do find flat plateaus instead of clearly defined energy minima for the open receptor states in our US calculations, presumably because only a LBD monomer is simulated and therefore able to open wider than a tetrameric complex. In an oligomer, a LBD opening beyond 120 degrees would begin to collide with other subunits leading to a rise in the free energy curve absent from our plots.

#### Ligand binding/dissociation

The second step of our description of ligand binding to the iGluR2 LBD involves removing the ligand from the open conformation of the receptor. This was conducted via US simulations in which we define a reaction coordinate as the center-of-mass distance between the ligand and a group of receptor amino acid residues deep within the binding site. Specifically, the backbone atoms of residues 399, 448–452, 462–464,476–481 and 705 were selected, resulting in a distance definition that ensures that the ligand is pushed out of the binding site when moving along the reaction coordinate. Receptor starting structures were based on the third minima (corresponding to the open structure) of the US receptor opening simulations described above.

The LBD was initially placed so that the two domains lie atop of each other along the z-axis and the reaction coordinate distance was measured in the XY-plane bisecting the binding cleft. The planar projection of the ligand position was merely used to define the position along the reaction coordinate. The ligand was fully free to move in all directions, but US biasing potentials acted on the XY-projection of the ligand position only, to ensure easy up and down movement of the ligands between the two receptor interfaces. LBD rotation and translation were removed every 10 MD steps. No additional orientational restraints were used on the system, with the exception of the US biasing potential, initially set to 600 kcal mol^−1^ nm^−2^, defining 23 windows spaced ∼0.2 nm apart from a starting distance of 0 nm to 3.5 nm. The average structure of the closed receptor complexes from MD simulations amount to corresponding protein-ligand distances on our reaction coordinate of 0.58 nm (AMPA) and 0.28 nm (BTA). These distance are slightly larger than the minima found due to the conformational changes of the recpetor.

As before, 300 ns length MD simulations were conducted for each window. For several US windows, the biasing harmonic potential force constant had to be increased to 1000 kcal mol^−1^ nm^−2^ to ensure that the system stayed close to its starting conformation. An initial analysis of histogram overlap indicated insufficient convergence of the free energy curves (data not shown) so an additional 22 US windows were added evenly spaced along the reaction coordinate. With additional data from these simulations, histogram overlap increased significantly and converged simulation curves were obtained.

**Figure 7:**
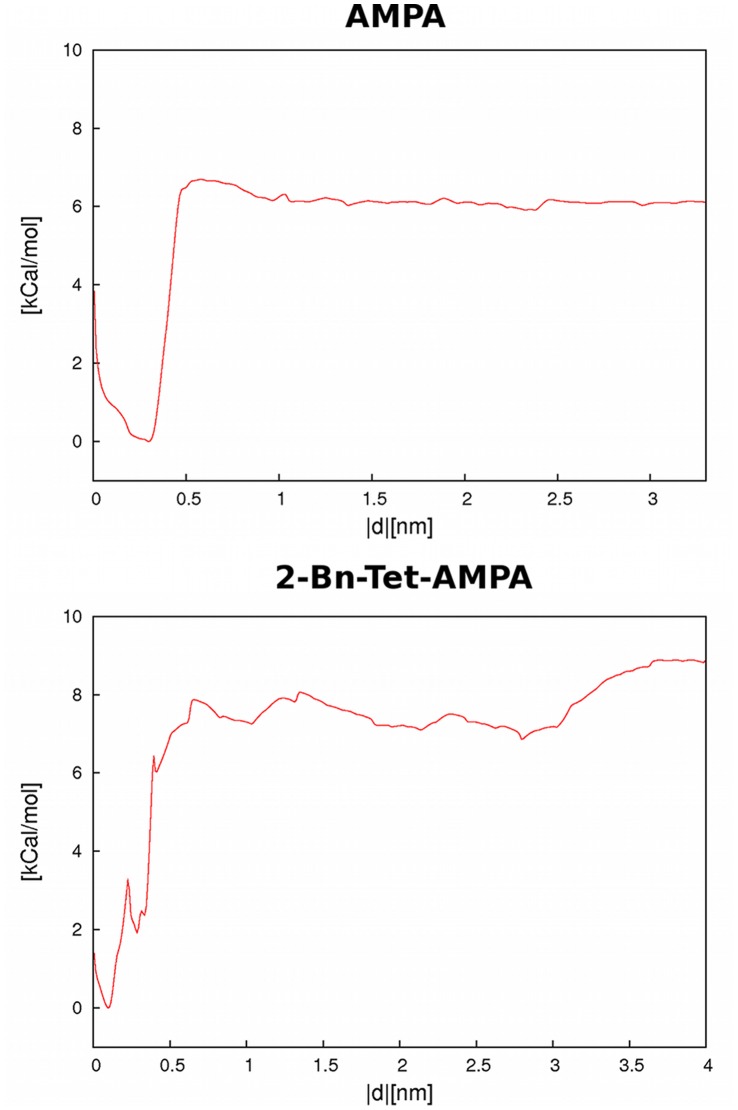
PMF free energy curves for the removal of the ligand from the LBD. The free energy curves correspond to data from 45 US windows using a total of 300 ns simulation length each. Depicted are the curves for the AMPA complex (top) and BTA complex (bottom). The reaction coordinate is defined as center-of-mass distance of the ligand and a group of protein residues, see text for details. For both simulations, a minimum corresponding to the tightly bound ligand at below 0.5 nm is found. During the unbinding process, we see a sharp rise of the free energy curve and a plateau at distances above 0.8 nm.

The PMF free energy curves for pushing AMPA and BTA out of the open LBD show fairly comparable free energy curves with a minimum at short distances and a significant energy barrier to overcome before ligands dissociate from the LBD (see Figure 7, only the final converged curves after 300 ns are shown). The minimum lies at a closer distance for BTA due to the different center-of-mass positions of the molecular structures, both represent tightly bound ligands. The barriers occur early in the unbinding process and after 0.5 nm an almost flat free energy curve outwards to 3 nm is found. This indicates that the LBD surface does not funnel ligands towards their binding position, instead ligands can randomly diffuse within the binding cleft until they reach a position very close to the minimum energy binding mode. Both free energy curves show few pronounced subminima and other structural features compared to the receptor opening process, only an intermediate state for BTA at 0.4 nm distance is suggested which could be due to random noise. In general, the BTA curve is less smooth and does not show a pronounced energy barrier at ca. 0.8 nm as for AMPA. It also shows considerable fluctuations at higher distances, again introducing some uncertainty when calculating the binding energy. BTA is a larger and more flexible ligand that is expected to take longer to converge in simulations.

Free energy costs to dissociate ligands from the LBD of 6.10 and 8.84 kcal/mol are obtained for AMPA and BTA, respectively. As for the receptor opening before, slightly larger values (indicating tighter binding) are found for BTA, but also a higher uncertainty of the results.

Summarily, the following picture emerges for the binding process of both ligands: Combining data from both simulated substeps, we obtain a total binding free energy from US calculations of −12.3 and −17.5 kcal/mol for AMPA and BTA respectively. Note that this result is in principle independent of our two-substep model, as converged free energy calculations should give the same total free energy change for all possible pathways. In practice, this is limited to low energy barrier pathways only. Nevertheless, converged free energy calculations will yield the same values for path-independent variables like binding free energies along any chosen path. While the process of receptor opening and ligand binding as simulated describes an intuitively reasonable complex formation/dissociation kinetics, the natural ligand binding may involve different elementary reactions. The fact that our two-step binding model leads to reasonable energy barriers for the overall process supports the model, but does not rule out other possible pathways.

From comparing the substep results, we see that the bound ligand has a significant influence on the LBD opening potential energy curve. AMPA results in a much tighter closure of the LBD, even though the closed receptor conformation is not noticeably different for the two complexes. This indicates strong interactions of AMPA with both domains on either side of the binding cleft surface. The AMPA-domain 1 interaction appears to be the dominant one, because the ligand remains attached to that domain during the LBD opening process. The free energy curve for receptor opening is significantly altered compared to the apo-case when AMPA is present. In this case AMPA-domain 2 interactions must be broken during opening, but AMPA-domain 1 interaction remain intact. This indicates strong interactions of AMPA with both domains.

The BTA ligand seems to bind comparably strong to both the closed and half-closed conformations of the receptor, since the difference of the two corresponding free energy minima are quite small (1.35 kcal/mol) and the barrier of ca. 2 kcal/mol can be overcame easily at room temperature, while for AMPA a clear preference of the closed state is found.

### Metadynamics

As an alternative to the extremely computationally expensive US simulations described above, we have conducted Metadynamics simulations in order to obtain the free energy surface for the LBD opening of the AMPA and BTA complexes (substep 1 in the US calculations above). We have chosen the three dominant eigenvectors from the rigid body PCA calculations above as the collective variables (CV1–CV3, namely clamshell motion, twisting motion and rocking motion) to be sampled.

From initial analysis of 50 ns simulations building the biasing function from 0.5 kcal/mol height Gaussian hills, added every picosecond, we observed significant convergence problems related to domain closure. The complex efficiently explored the phase space along all collective variables, but the system nevertheless did not return to a conformation close to the closed complex starting state. We then refined our protocol to build the biasing potential slower, adding 0.2 kcal/mol energy hills every 10 ps for 200 ns, followed by 0.05 kcal/mol energy hills, leading to converged potential energy landscapes.

500 ns Metadynamics simulations starting from the closed receptor form with bound AMPA or BTA were conducted. The simulations for BTA were extended another 100 ns to ensure convergence, but no significant changes in the free energy surface were found. The receptor opening and closing requires motion along the clamshell and twisting modes for both complexes. CV3 is less important to describe the potential energy surface for the AMPA complex, as all observed minima and saddle points lay in approximately the same CV3 coordinate range and the simulations rarely deviated from it. In contrast, for BTA, the rocking motion CV3 is an important part of the opening process as well.

**Figure 8:**
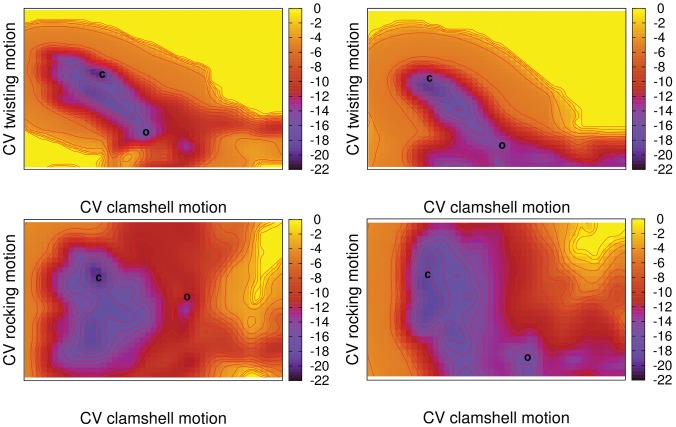
Potential energy surface projected onto the first, second and third collective variables for the opening/closing transition of the AMPA complex. Data from 500 ns of Metadynamics simulations is plotted for each complex. The two minima corresponding to the open and closed state of the LBD are indicated.

**Figure 9:**
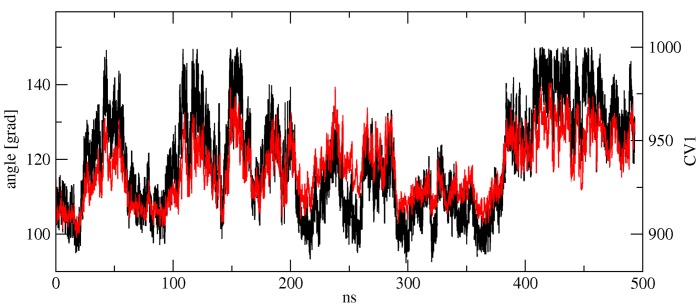
Comparison of the time evolution of the interdomain angle (black) and main collective variable CV1 (red) over the course of a 500 ns length Metadynamics simulation. Both geometric parameters show very similar trends, indicating that the choice of CV for Metadynamics yields a meaningful exploration for functional interdomain motions.

Plotting the PES along these variables (see Figure 8) shows a two-state system with minima corresponding to the open and closed state indicated. No indication of the local minimum for the half-closed state can be discerned. The open state is predicted to lie 4.30 kcal/mol above the closed state for AMPA and 4.86 kcal/mol for BTA in reasonable agreement to the US calculations above. During the course of the simulation, we observe multiple transitions between the two states, highlighting the improvement in sampling the Metadynamics simulations provide. A comparison of the time evolution of the interdomain angle and the main collective variable (see Figure 9) over the course of the Metadynamics simulation shows very similar trends for both geometric parameters, indicating that the choice of CV yields a meaningful exploration of the domain opening/closing motion.

It appears that with the additional biasing potentials added during the Metadynamics run, a converged sampling of this two-state system is possible during realistic simulation times at a much lower cost than for the US curves (where each of many simulation windows used hundreds of nanoseconds simulations time).

## Discussion

We show that the iGluR2 LBD remains stable over unprecedented length of MD simulations. Both the closed (complexed) and open (apo-) forms of the receptor maintain their conformational state over the course of 200 ns. The open, ligand-free form is able to open wider than known from X-ray structures, presumably due to the absence of interdomain interactions in the simulated monomer LBD. This indicates that in the complete receptor, opening the clamshell binding cleft beyond 120 degrees is a restricted motion that could initiate functional conformational changes connected to channel opening or cause allosteric effects. When ligands are bound in the closed receptor form, their binding modes remain stable over hundreds of nanoseconds. Ligand binding modes in very good agreement to experimental results are found, however the amount of binding site solvation by structural water molecules is reduced for the AMPA ligand. Here, water mediated H-bonds are replaced by direct ones. The cause for this could lie in an overestimation of directed, electrostatic interaction energies between ligand and protein in the force field used. Another explanation is that a description of bound water molecules in a low dielectric region like a protein interior is difficult to accomplish for fixed charge force fields. All tested water models were parameterized for the liquid water state and are generally overpolarized to account for water-water interactions. An improved description of intra-binding site water molecules may require using a polarizable or even quantum mechanical water model. We are currently investigating this effect in further detail for the case of the iGluR2 LBD.

The US free energy calculations for the opening/closing of the LBD yield converged PMF curves after simulation times of more than 100 ns per US window. Even after 300 ns, small changes in the free energy curve are still obtained. This reinforces the typical case of studying large scale conformational changes of biomolecules, even after conducting more than a total of 5 microseconds of computer simulations, perfect convergence is not reached. Nevertheless, we can clearly identify the LBD clamshell as a three state system. It contains the closed state, the most stable conformation when a ligand is bound, the half-closed state typical for partial agonist ligands, which is only evident in the presence of a bound ligand, and an open state that is the most stable form of the apo-protein. In good agreement to the free MD simulations above, the open receptor state corresponds to a very shallow energy minimum or plateau in the free energy curve, presumably caused by the lack of interaction with other domains in our monomer simulations. A comparison of the two ligand complexes shows that the AMPA ligand stabilizes the closed form much stronger than BTA. For BTA, the closed and half-closed form appear to be comparable in energy. Still, it is known that bound BTA causes the LBD to close fully and it shows typical agonistic activity [9].

Removing the ligands from the open receptor forms yields very comparable curves for both ligands, however a slightly higher binding strength is found for BTA. We find that the ligands need to be comparably close to their final binding positions to experience stabilisation from the protein.

The overall results and the suggested binding model agree well with other simulation studies of the functional conformational changes in bilobal receptors. One example of an unrelated but structurally similar system is the ribose-binding protein (RBP) from the family of bacterial periplasmic receptors. For RBP, a pioneering umbrella sampling study by Ravindranathan et al. could identify a ligand induced shift in the equilibrium between an open and a closed form of the receptor [59]. Similarly to the case here, for the ligand-free receptor an open structure dominates the conformational ensemble, which upon ligand binding shifts to strongly favor the closed form. Interestingly, for the RBP, a half-closed structure, similar to the partial agonist bound structure for iGluR2, was found as well. It appears that for cases where the open and closed end state structures are available from X-ray crystallography, US simulations are capable to describe even very large scale conformational changes in receptor proteins accurately, though at very high computational cost.

The Metadynamics approach to free energy estimation yielded converged PES maps for the opening of the LBD complexes after using a refined protocol. It appears that sampling along the CV coordinate system is very effective, even though there are many additional relevant degrees of freedom in a macromolecular complex. Metadynamics has been successfully applied to macromolecular binding phenomena before [55]. The sampling efficiency of the approach depends in non-trivial ways on simulation length, selection of CV and other adjustable parameters. It is possible that different CV selections or a modified protocol for the addition of biasing potentials would result in a better converging simulation. Since results seem to be somewhat parameter dependent, we conclude that the Metadynamics approach can be problematic to apply to a ligand binding system with a narrow receptor binding cleft, even though it was possible in the present case. The method remains highly appealing in principle due to its reduced simulation cost and unrestrained exploration of conformational space when compared to US simulations, but for the present case, where a plausible reaction coordinate can be defined (since the end states of the binding process are known from X-ray structure analysis), US free energy calculations remain a reliable computational tool. We propose that future studies could employ Metadynamics results in a similar way as done here: If a single well-defined reaction coordinate is available, extensive umbrella sampling calculations along this degree of freedom can establish a validation PMF. Then, Metadynamics simulations are conducted using the US reaction coordinate as one collective variable. This allows the Metadynamics simulation to explore the complex conformational energy landscape, while the free energy profile along at least one CV can be compared to a highly reliable alternative data set. This way extreme costly multidimensional US calculations are avoided but all relevant degrees of freedom are sampled.

The Metadynamics simulations yielded slightly smaller free energies for the opening process of the LBD. A comparison to the US values gives an indication that the uncertainty due to insufficient sampling for both approaches is more than 1 kcal/mol for the AMPA case and more than 3 kcal/mol for BTA. Especially for the latter ligand, a large part of the uncertainty comes from the more noisy free energy curves that make determining the energies of the unbound or open states difficult. It is unfortunate but not unexpected that sampling problems occur especially for these flexible states that require sampling of extremely large phase space regions, while the parts of the free energy curves corresponding to the compact, bound states are much better defined.

Combining the data and error estimate above, assuming the unbinding step II to have comparable accuracy to the domain opening step I and averaging the free energy results from US and Metadynamics for step I, we obtain predicted binding free energies of 11.3±2 kcal/mol and 15.6±6 kcal/mol for AMPA and BTA (Free Energy values summarized in Table 2). For AMPA, this result is in excellent agreement to previous calculation in the pioneering work of Roux et al. [36] and very close to the experimental value of −10.8 kcal/mol. For BTA, the larger error estimate makes a comparison more difficult. An experimental affinity value in the low micromolar range from affinitiy data in Ref. [60] is close to the lower range of the estimated binding strength, but our calculations definitely overestimate the binding strength of BTA somewhat.

**Table 2:** Free Energy differences calculated by Umbrella Sampling and Metadynamics (in kcal/mol).

	AMPA	BTA
US Step1	6.18	8.69
US Step2	6.10	8.84
US Σ	12.28	17.53
Metadynamics	4.30	4.86

Free Energy differences were calculated by taking the differences of the Free Energy values at the corresponding minima in the free energy curve. Metadynamics results correspond to the “US Step 1” calculations.

The free energy calculations presented in this work do support the model of ligand induced shifts in the receptor conformational ensemble. In this case, A ligand-free receptor existing mainly in the open conformation will be induced to almost exclusively occupy the closed state after ligand binding. However, while our simulations show that the two step process of ligand binding to the open receptor followed by closing of the complex is feasible, it does not rule out other possible pathways. Our free energy estimates for the end states should be valid independent of the reaction coordinate, but this implies sufficient sampling of all parts of the reaction coordinate. The very extensive length of the MD simulations presented makes sufficent sampling likely in this case, but as for all MD based studies, this can not be proven. However, previous US studies of similar systems have produced converged free energy results with significantly shorter simulations.

The accuracy of the free energy results was estimated to be in the range of few kcal/mol above. This would not be considered exceptional accuracy for free energy calculations on small molecules, but is in the expected range for large macromolecular changes. The good agreement to experimental values where available indicates that additional sources of error, such as force field inaccuracies, neglect of polarisation in a fixed charge model and finite sampling are either small or tend to compensate each other.

Overall, we show that computer simulations offer a detailed picture of iGluR2-ligand interactions and provide a consistent two-step mechanism for the binding and activation process. We show that the BTA ligand binds fairly similar to AMPA, suggesting potential future studies of iGluR2 activation and control via photo-switchable new compounds based on BTA. For future studies, the binding of such novel ligands could be studied as described here, with the additional degree of freedom of the induced ligand conformational change while bound to the receptor. Free Energy calculations to determine the shift in the structural ensemble when a ligand bound in the closed complex undergoes a conformational change could shed light on the atomistic details of photo-switching a receptor. Additionally, the model of a single two-domain receptor should be extended to the full oligomeric complex, including the membrane bound ion channel. Simulations of the full assembly could describe cooperative effects and explain the signal transduction from ligand binding to channel opening.

## Supporting Information

Figure S1 **Measuring Interdomain Motions for iGluR during long MD simulations.** To better understand the connection between the different receptor states and the corresponding interdomain geometry, we have measured the interdomain angle (the LBD opening motion) over the course of several long MD simulations. The geometry definition is the same as used in the rigid PCA analysis. For the interdomain angle this was the angle between domain 1 and 2 centers of mass (defined as residues 394–495, 732–771 for domain 1 and residues 500–728 for domain 2) measured from the center of mass of the flexible hinge region (defined as residues 496–499 and 729–731). This geometric parameter was measured over the course of nine 200 to 300 ns length MD simulations. We compare three simulations starting from X-ray crystal structures, one with the bound agonist AMPA (pdb entries 1FTM), one with the bound partial agonist IW (pdb entries 1MQG) and one of the apo-protein (pdb entries 1FTO). In addition, we analyzed umbrella sampling simulation windows corresponding to the three minima in the PMF free energy curve both for the AMPA and BTA receptor complexes (see main text for model details). It can be seen that for each umbrella sampling window, the geometry parameters are close to those of the corresponding X-ray crystal structure simulation. Furthermore, for the apo-protein geometric parameters in general fluctuate more, indicating the more flexible nature of this structure. The interdomain angles for the closed structures do not change very much over the course of the MD simulations, indicating the direct connection between the interdomain angle and the opened/closed state of the receptor.
(TIFF)

Figure S2 **Measuring Interdomain Motions for iGluR during long MD simulations.** To better understand the connection between the different receptor states and the corresponding interdomain geometry, we have measured the interdomain torsion (the twisting motion) over the course of several long MD simulations. The geometry definition is the same as used in the rigid PCA analysis. For the interdomain torsion, the dihedral was defined using the same domain definitions as for the interdomain angle above for the torsion end points and the upper and lower parts of the hinge region as the rotateable axis (defining residues 498 and 730 as one end of the hinge region and residues 499 and 729 as the second one). This geometric parameter was measured over the course of nine 200 to 300 ns length MD simulations. We compare three simulations starting from X-ray crystal structures, one with the bound agonist AMPA (pdb entries 1FTM), one with the bound partial agonist IW (pdb entries 1MQG) and one of the apo-protein (pdb entries 1FTO). In addition, we analyzed umbrella sampling simulation windows corresponding to the three minima in the PMF free energy curve both for the AMPA and BTA receptor complexes (see main text for model details). It can be seen that for each umbrella sampling window, the geometry parameters are close to those of the corresponding X-ray crystal structure simulation. Furthermore, for the apo-protein geometric parameters in general fluctuate more, indicating the more flexible nature of this structure. In contrast to the interdomain angle, the interdomain torsion is less crucial for opening closing and can be seen to change more frequently, even for closed structures.
(TIFF)

Movie S1 **First eigenvector of iGlu2 receptor conformational motion.**
(MPG)

Movie S2 **Second eigenvector of iGlu2 receptor conformational motion.**
(MPG)

Movie S3 **Third eigenvector of iGlu2 receptor conformational motion.**
(MPG)
