# Correction: Computational Study of Synthetic Agonist Ligands of Ionotropic Glutamate Receptors

**DOI:** 10.1371/annotation/41544025-f07e-49bc-84f1-3d8aa3130db1

**Published:** 2014-01-17

**Authors:** Tino Wolter, Thomas Steinbrecher, Marcus Elstner

Publication was supported by Deutsche Forschungsgemeinschaft and Open Access Publishing Fund of Karlsruhe Institute of Technology.

