# Supplementary figures and images for: Computational Study of Synthetic Agonist Ligands of Ionotropic Glutamate Receptors
Source: PLoS One. 2013 Mar 25;8(3):e58774. doi: 10.1371/journal.pone.0058774 (PMC3607592; doi:10.1371/journal.pone.0058774)

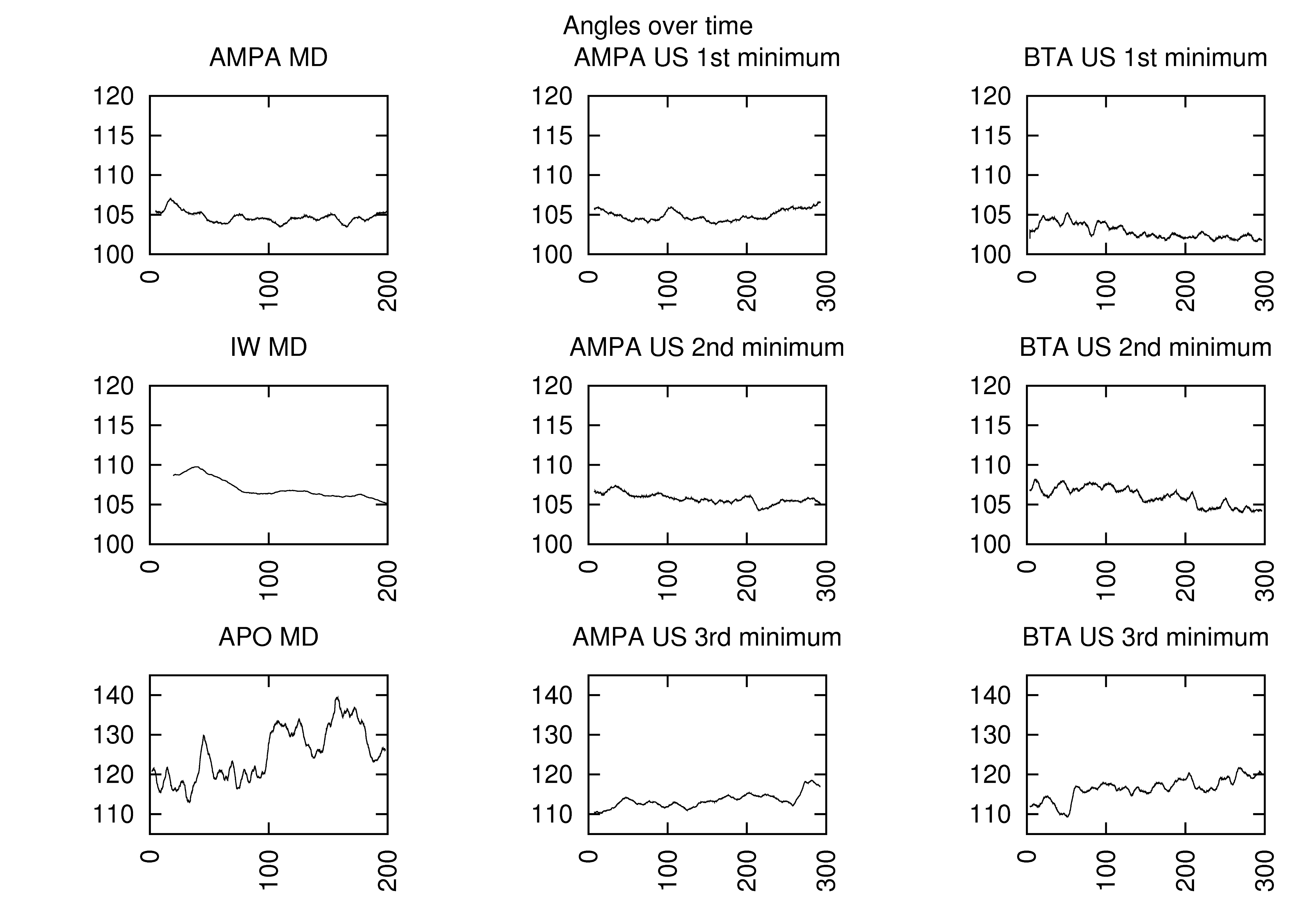

Supplement: Figure S1 — Measuring Interdomain Motions for iGluR during long MD simulations. To better understand the connection between the different receptor states and the corresponding interdomain geometry, we have measured the interdomain angle (the LBD opening motion) over the course of several long MD simulations. The geometry definition is the same as used in the rigid PCA analysis. For the interdomain angle this was the angle between domain 1 and 2 centers of mass (defined as residues 394–495, 732–771 for domain 1 and residues 500–728 for domain 2) measured from the center of mass of the flexible hinge region (defined as residues 496–499 and 729–731). This geometric parameter was measured over the course of nine 200 to 300 ns length MD simulations. We compare three simulations starting from X-ray crystal structures, one with the bound agonist AMPA (pdb entries 1FTM), one with the bound partial agonist IW (pdb entries 1MQG) and one of the apo-protein (pdb entries 1FTO). In addition, we analyzed umbrella sampling simulation windows corresponding to the three minima in the PMF free energy curve both for the AMPA and BTA receptor complexes (see main text for model details). It can be seen that for each umbrella sampling window, the geometry parameters are close to those of the corresponding X-ray crystal structure simulation. Furthermore, for the apo-protein geometric parameters in general fluctuate more, indicating the more flexible nature of this structure. The interdomain angles for the closed structures do not change very much over the course of the MD simulations, indicating the direct connection between the interdomain angle and the opened/closed state of the receptor. (TIFF) [file pone.0058774.s001.tif]

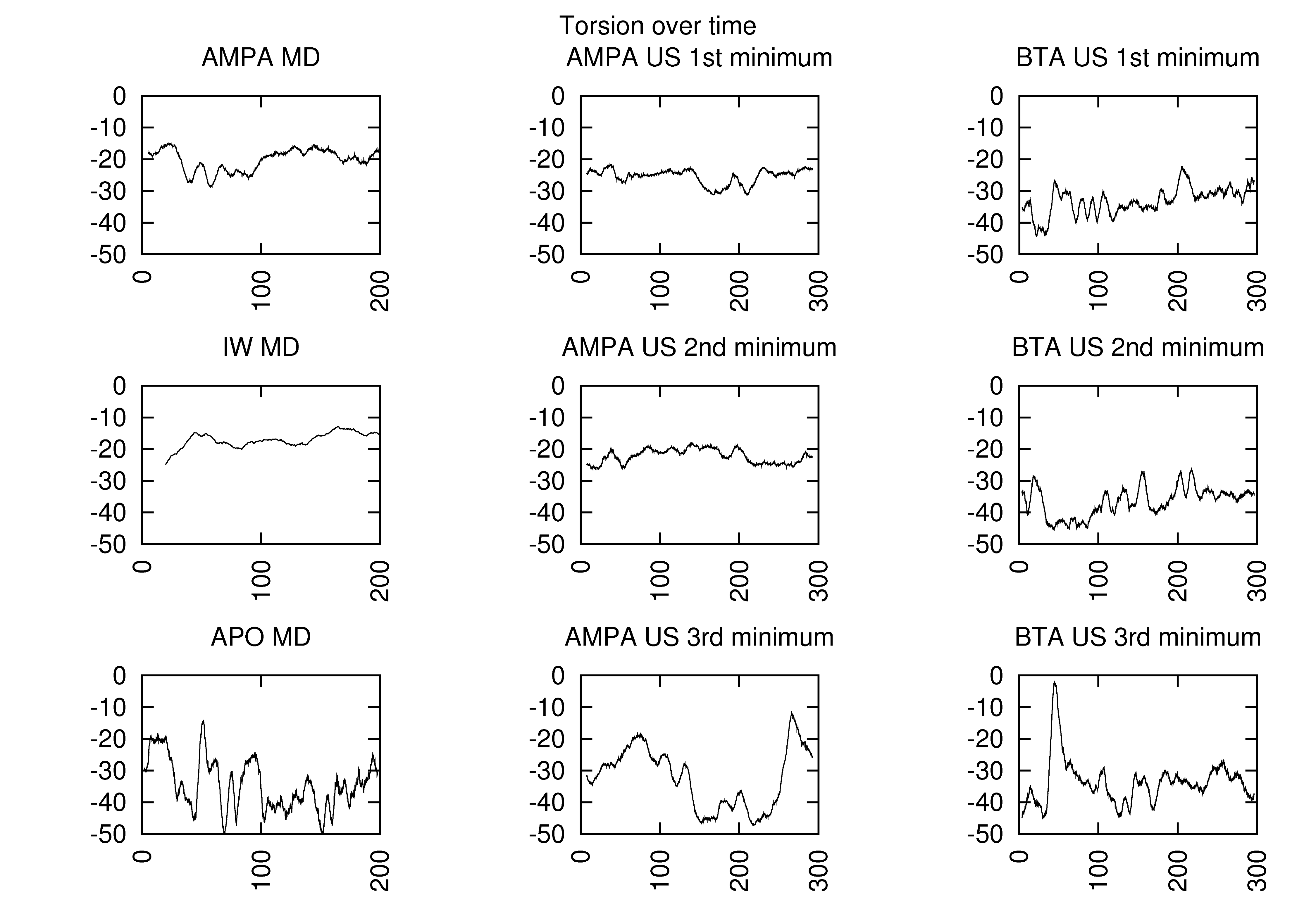

Supplement: Figure S2 — Measuring Interdomain Motions for iGluR during long MD simulations. To better understand the connection between the different receptor states and the corresponding interdomain geometry, we have measured the interdomain torsion (the twisting motion) over the course of several long MD simulations. The geometry definition is the same as used in the rigid PCA analysis. For the interdomain torsion, the dihedral was defined using the same domain definitions as for the interdomain angle above for the torsion end points and the upper and lower parts of the hinge region as the rotateable axis (defining residues 498 and 730 as one end of the hinge region and residues 499 and 729 as the second one). This geometric parameter was measured over the course of nine 200 to 300 ns length MD simulations. We compare three simulations starting from X-ray crystal structures, one with the bound agonist AMPA (pdb entries 1FTM), one with the bound partial agonist IW (pdb entries 1MQG) and one of the apo-protein (pdb entries 1FTO). In addition, we analyzed umbrella sampling simulation windows corresponding to the three minima in the PMF free energy curve both for the AMPA and BTA receptor complexes (see main text for model details). It can be seen that for each umbrella sampling window, the geometry parameters are close to those of the corresponding X-ray crystal structure simulation. Furthermore, for the apo-protein geometric parameters in general fluctuate more, indicating the more flexible nature of this structure. In contrast to the interdomain angle, the interdomain torsion is less crucial for opening closing and can be seen to change more frequently, even for closed structures. (TIFF) [file pone.0058774.s002.tif]
